# Sex-specific interaction between cortisol and striato-limbic responses to psychosocial stress

**DOI:** 10.1093/scan/nsab062

**Published:** 2021-04-16

**Authors:** Gina-Isabelle Henze, Julian Konzok, Ludwig Kreuzpointner, Christoph Bärtl, Marina Giglberger, Hannah Peter, Fabian Streit, Brigitte M Kudielka, Peter Kirsch, Stefan Wüst

**Affiliations:** Department of Psychology, Institute of Psychology, University of Regensburg, Regensburg 93053, Germany; Department of Psychology, Institute of Psychology, University of Regensburg, Regensburg 93053, Germany; Department of Psychology, Institute of Psychology, University of Regensburg, Regensburg 93053, Germany; Department of Psychology, Institute of Psychology, University of Regensburg, Regensburg 93053, Germany; Department of Psychology, Institute of Psychology, University of Regensburg, Regensburg 93053, Germany; Department of Psychology, Institute of Psychology, University of Regensburg, Regensburg 93053, Germany; Department of Genetic Epidemiology in Psychiatry, Central Institute of Mental Health, Medical Faculty Mannheim, Heidelberg University, Mannheim 68159, Germany; Department of Psychology, Institute of Psychology, University of Regensburg, Regensburg 93053, Germany; Department of Clinical Psychology, Central Institute of Mental Health, Medical Faculty Mannheim, Heidelberg University, Mannheim 68159, Germany; Department of Psychology, Institute of Psychology, Heidelberg University, Heidelberg 69047, Germany; Department of Psychology, Institute of Psychology, University of Regensburg, Regensburg 93053, Germany

**Keywords:** hippocampus, amygdala, nucleus. caudatus, nucleus. accumbens, thalamus, fMRI

## Abstract

Although women and men differ in psychological and endocrine stress responses as well as in the prevalence rates of stress-related disorders, knowledge on sex differences regarding stress regulation in the brain is scarce. Therefore, we performed an in-depth analysis of data from 67 healthy participants (31 women, taking oral contraceptives), who were exposed to the Scan*STRESS* paradigm in a functional magnetic resonance imaging study. Changes in cortisol, affect, heart rate and neural activation in response to psychosocial stress were examined in women and men as well as potential sex-specific interactions between stress response domains. Stress exposure led to significant cortisol increases, with men exhibiting higher levels than women. Depending on sex, cortisol elevations were differently associated with stress-related responses in striato-limbic structures: higher increases were associated with activations in men but with deactivations in women. Regarding affect or heart rate responses, no sex differences emerged. Although women and men differ in their overall stress reactivity, our findings do not support the idea of distinct neural networks as the base of this difference. Instead, we found differential stress reactions for women and men in identical structures. We propose considering quantitative predictors such as sex-specific cortisol increases when exploring neural response differences of women and men.

## Introduction

On average, women and men show various differences in variables related to the central nervous system (CNS), including neuroanatomical, autonomic and psychological variables; consistently, a sex-specific genetic architecture was found for several CNS-related phenotypes (McCarthy *et al.*, 2017; David *et al.*, 2018). With the advent of human brain–imaging techniques, sex differences in the brain were found, covering—among others—anatomical variables, connectivity measures, as well as neural correlates in affect and cognitive functions (Cahill, 2006; Ingalhalikar *et al.*, 2014a; Grabowska, 2017; Choleris *et al.*, 2018; Ritchie *et al.*, 2018). These findings are paralleled by clear sex differences in prevalence rates for stress-related mental disorders (Kudielka and Kirschbaum, 2005; Bangasser and Valentino, 2014). On the other hand, the distribution of variables such as functional connectivity (FC) or neuroanatomical variables was also reported to be overlapping in women and men (Joel *et al.*, 2015, 2018, 2020; Joel and Fausto-Sterling, 2016; Grabowska, 2017). Therefore, it was assumed that, depending on methodological aspects, distinct sexual dimorphisms may or may not be detectable in neural variables (Joel and Tarrasch, 2014; Ingalhalikar *et al.*, 2014b; Del Giudice *et al.*, 2015, 2016; Chekroud *et al.*, 2016; Rosenblatt, 2016). This leads to the plausible and relevant question as to what extent sex differences in stress regulation and stress-related psychopathology can be attributed to differences between women and men in the brain’s response to stress.

Women tend to report more perceived stress, anxiety and tension during and after acute stress exposure than men (Kirschbaum *et al.*, 1999; Kelly *et al.*, 2008; Merz and Wolf, 2015). Interestingly, these self-report–based differences are consistent with findings in animal models, as female rodents show more passive and stress-related behaviors in response to stress (Beery and Kaufer, 2015; McEwen and Milner, 2017; Rincón-Cortés *et al.*, 2019). Moreover, mean corticosterone stress responses are higher in female than in male rodents (Goel *et al.*, 2014; Oyola and Handa, 2017). However, sex differences in hypothalamic–pituitary–adrenal (HPA) axis reactivity in rodents are not consistent with findings in humans, as healthy men show significantly larger responses than women to acute psychosocial stress induction (Kudielka and Kirschbaum, 2005; Liu *et al.*, 2017; Zänkert *et al.*, 2019). The reasons for this inconsistency between animal and human studies are not well understood, but significant differences in both stress quality and intensity could hamper the comparability of findings. While animals are often exposed to completely novel, uncontrollable and potentially life-threatening aversive situations, human participants usually experience moderate social-evaluative threat while being fully aware that they can abort the exposure at any time. Moreover, possible explanations for this sex-specific response pattern in humans range from an impact of stressor type (social rejection challenges as typical female stressors, achievement stressors as typical male stressors) to a modulating impact of hormonal concentration fluctuations in different menstrual cycle phases and the use of oral contraceptives (OCs; Zänkert *et al.*, 2019). Regarding heart rate responses to stress, findings are more inconclusive, with some studies reporting sex differences (Seo *et al.*, 2017; Emery *et al.*, 2018) that partly depended on menstrual cycle phases (Kudielka *et al.*, 2004; Childs *et al.*, 2010), while others failed to find differences (Kirschbaum *et al.*, 1999; Kelly *et al.*, 2008).

So far, a few attempts have been made to evaluate neural sex differences by implementing distinct stress paradigms in functional magnetic resonance imaging (fMRI) environments. A perfusion-based fMRI study reported a sex-specific neural activation model featuring primarily striato-limbic activation in women and asymmetric frontal blood flow in men. Moreover, the correlation between these sex-specific activation patterns and cortisol was higher in men (Wang *et al.*, 2007). Regarding neural stress processing, (pre)limbic structures seem to be of particular relevance since dissociations between women and men for these regions have been reported (Seo *et al.*, 2011, 2017; Kogler *et al.*, 2015; Goldfarb *et al.*, 2019). Focusing on the amygdala, resting state FC studies emphasized sex-specific responses in limbic circuits. Furthermore, associations between FC and cortisol were also found to differ significantly between women and men (Henckens *et al.*, 2010; Veer *et al.*, 2012; Vaisvaser *et al.*, 2013; Vogel *et al.*, 2015; Kogler *et al.*, 2016). To date, investigations into sex differences regarding the neural processing of psychosocial stress remain scarce and yielded mixed results (Noack *et al.*, 2019). While some evidence for stress-induced neural response differences between women and men exists (Kogler *et al.*, 2015, 2017; Chung *et al.*, 2016a; Dahm *et al.*, 2017), no consistent sex-specific neural response pattern emerged.

The heterogeneity of previous findings can probably—at least in part—be explained by methodological disparities resulting from different stress induction paradigms. Studies varied in dependent variables (endocrine, subjective and cardiovascular) as well as in stress intensity and thereby in the magnitude of stress responses (Noack *et al.*, 2019). A recent study by our group (Henze *et al.*, 2020) aimed at elucidating the interaction of distinct stress activation systems in response to an improved psychosocial fMRI stress protocol (Streit *et al.*, 2014). We found significant cortisol, subjective, heart rate and neural reactions in response to Scan*STRESS* (Henze *et al.*, 2020). Moreover, neural stress reactions in (pre)limbic structures were associated with individual changes in cortisol and negative affect ratings. Given the robust cortisol responses and the consistent interactions between different stress response domains within a relatively large cohort, it appeared promising to further elaborate the role of sex within the same sample. Therefore, the objective of the present study was a detailed and comprehensive analysis of sex-related stress response differences in distinct response domains including neural and cortisol responses as well as changes in heart rate and affect. Instead of merely contrasting female and male responses dichotomously, as most of the studies on sex differences in neural stress processing did (Kogler *et al.*, 2015, 2017; Chung *et al.*, 2016a; Dahm *et al.*, 2017), we applied a statistical model including individual cortisol increases as a continuous predictor to examine stress-related sex differences in the brain (Joel and Fausto-Sterling, 2016; Grabowska, 2017; Joel *et al.*, 2018). This model assumed that sex differences in neural stress processing might emerge when sex-related cortisol response differences are considered.

As an exploratory analysis, we also studied whether dynamic changes in neural stress responses, previously reported for (pre)limbic structures (Henze *et al.*, 2020), differ between women and men. Assuming that this exposure-time effect may indicate sensitization processes of ongoing stress exposure, sex differences might corroborate to a better understanding of interindividual differences related to stress vulnerability. For instance, in animals, chronic stress was found to cause damages to the hippocampus in male rats and monkeys but less, if at all, in females (McEwen, 2000).

## Material and methods

### Participants

Sixty-seven young, healthy, scanner-naïve university students (mean age 23.06 ± 3.14 years) participated in the present study. Stress-induced cortisol, affect, heart rate and neural responses of the present sample have been previously reported (Henze *et al.*, 2020). It consisted of 31 women (mean age 22.10 ± 2.12 years) and 36 men (mean age 23.89 ± 3.64 years). Owing to HPA axis activity differences depending on menstrual cycle phase and OC use (Kudielka and Kirschbaum, 2005; Zänkert *et al.*, 2019), only women using OCs were tested. Participants were recruited via flyers and social media internet platforms. Individuals who met any of the following criteria were excluded: self-reported history of or current psychiatric, neurological or endocrine disorders; treatment with psychotropic medications or other medication affecting CNS or endocrine functions; daily tobacco or alcohol use; incompatibility with fMRI scanning (e.g. metal parts and pregnancy); regular night-shift work and undergoing a current stressful episode (e.g. major exams or emotional stress due to separation from partner or serious illness/death of a family member). All participants provided written informed consent and received a monetary compensation. The study was approved by the local ethics committee of the University of Regensburg.

### General procedure and statistical analysis of cortisol, affect and heart rate data

**Fig. 1.**
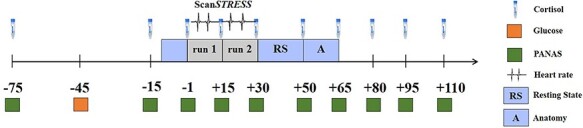
Experimental procedure including the repeated collection of cortisol samples, affect ratings and heart rate measurements during Scan*STRESS.*

To induce psychosocial stress in the fMRI environment, the Scan*STRESS* paradigm was applied (Streit *et al.*, 2014); see Supplementary Methods A.1 and Figure SA1. Briefly, Scan*STRESS* is composed of an alternating block design in a fixed order, presented in two runs, containing two conditions (stress *vs* control) prompting the participants to perform arithmetic and rotation tasks while a feedback-giving observation panel is presented via live video stream providing disapproving feedback. Moreover, between the two runs, the participants are notified that their performance was below average and they have to improve in the second run. For the present study, the protocol was slightly modified without changing the paradigm itself, in particular to improve cortisol responses to stress induction in the fMRI, as previous results were not always fully convincing (Streit *et al.*, 2014; Noack *et al.*, 2019). First, we implemented a prolonged (45 min) relaxing phase prior to stress to create sufficient baseline conditions (i.e. low cortisol levels). Moreover, we provided a detailed description and comprehensive clarification about the general scanning procedure (before the testing sessions took place) to minimize concerns prior to scanning that may confound with the response to the paradigm itself (McGlynn *et al.*, 2007; Thorpe *et al.*, 2008). Second, we administered a sugary drink (75 g glucose in 200 ml herbal tea) as it proved to facilitate cortisol reactivity (Gonzalez-Bono *et al.*, 2002; Zänkert *et al.*, 2020). The underlying mechanisms are still unknown but an influence of hunger and saturation regulating neuropeptides was discussed (Rohleder and Kirschbaum, 2007). Third, we achieved a more abrupt passage (<10 min) from relaxation to stress exposure (details see Henze *et al.* (2020)). Figure 1 provides an overview of the improved procedure. Test sessions took place between 1 and 6 PM.

Saliva samples for cortisol assessment were collected at 10 time points (−75, −15, −1, +15, +30, +50, +65, +80, +95, +110 min) using ‘Cortisol Salivettes’ (Sarstedt, Nuembrecht, Germany). To collect samples at minutes −1 to +65, the experimenter, wearing medical gloves, gave the Salivette swab to the participant lying in the scanner. Mood state was compiled at the same 10 time points using the German version of the Positive and Negative Affect Schedule scales (Watson *et al.*, 1988). Saliva samples were stored at −20°C until analysis. Samples were assayed in duplicate using a time-resolved fluorescence immunoassay with fluorometric end-point detection (dissociation-enhanced lanthanide fluorescence immunoassay) at the biochemical laboratory of the University of Trier (Dressendörfer *et al.*, 1992); see Supplemental Methods A.2 for details. The intra-assay coefficient of variation was between 4.0% and 6.7%; inter-assay coefficients of variation were between 7.1% and 9.0%. During Scan*STRESS*, heart rate recordings were obtained with an MRI-compatible finger oximeter (Model 7500 FO; Nonin Medical, Plymouth, MN) on the index finger, with a sampling rate of the highest heart beat within 4 s.

Data were analyzed in IBM SPSS Statistics version 25 (IBM, Corp., Armonk, NY) using repeated-measures analyses of variance (ANOVAs) regarding cortisol (nmol/l), positive and negative affect (test score) and heart rate (beats/min) with time as within-subjects factor and sex as between-subjects factor. A cortisol increase was defined as the difference between the individual cortisol peak (sample +30, +50 and +65) and the pre-stress cortisol level (sample −1). Cortisol responder rates were computed with an increase of at least 1.5 nmol/l rise being defined as response (Miller *et al.*, 2013). We used untransformed values for our analyses, as e.g. log-transformations were not necessarily appropriate for each measurement point. Therefore, we inspected skewness and kurtosis of time points individually (see Supplemental Methods A.3 for details) and found that they did not exceed acceptable values (Miles and Shevlin, 2001; Keele, 2008). Mean heart rates were calculated separately for each control and stress block. Greenhouse–Geisser corrections were applied where appropriate, and only adjusted results are reported.

### fMRI acquisition and data analysis

Participants were scanned in a Siemens MAGNETOM Prisma 3 T MRI (Siemens Healthcare, Erlangen, Germany) equipped with a 64-channel head coil. A series of blood-oxygenation-level-dependent gradient echo-planar-imaging (EPI) images was acquired with the following parameters: repetition time 2000 ms, echo time 30 ms, 90° flip angle, 64 × 64 matrix, 192 mm field of view and 37 3 mm axial slices with 1 mm gap. Data were analyzed using FSL 6.0 (FMRIB Software Library, Oxford, United Kingdom). The first five EPI volumes were discarded to allow for T1 equilibration. fMRI data processing was carried out using FEAT version 6.0 (see Supplemental Methods A.4 for details). The *z* (Gaussianized *t/F*) statistic images were thresholded nonparametrically using clusters determined by either *z* > 3.1 or *z* > 2.3.

For each subject, general linear models (GLMs) were defined containing regressors for control and stress conditions and the respective announcement phases. In sum, 12 regressors resulted: six conditions (stress arithmetic subtraction, stress figure rotation, control numbers, control figures, announcement of stress and announcement of control) and six motion regressors. GLMs were carried out on three levels: for each subject, one GLM was computed for each run (first level, *z* > 3.1) to account for scanner drifting. Subsequently, a fixed effects analysis (second level, *z* > 3.1) was obtained to measure mean responses. On a third level, unpaired two-group analyses (mixed effects, *z* > 2.3) were conducted to study sex differences: first, an unpaired two-group difference analysis was conducted to study sex-specific (men > women and women > men) responses for the main task effects, namely stress > control and control > stress. Second, we performed an unpaired two-group analysis with continuous covariate interaction (grand mean centered) to examine if the linear relationships between neural responses and cortisol increases (continuous covariate) differ between women and men (men > women and women > men). This model considers mean cortisol response differences between women and men. In addition, this model enabled to evaluate the interaction of cortisol and neural responses in the two subsamples separately. Corrections were performed over the whole brain with each contrast (stress > control, control > stress) thresholded at familywise error (FWE) *P* < 0.025 (two-tailed combined test, FWE *P* < 0.05).

Association analysis with cortisol increase was computed within a priori-defined striato-limbic anatomical regions of interest (ROIs) using masks from the Harvard-Oxford Atlas. We included the following eight masks, as the respective regions have been reported to respond to stress in a sex-specific manner (Wang *et al.*, 2007; Seo *et al.*, 2011, 2017; Kogler *et al.*, 2015, 2017; Chung *et al.*, 2016a; Dahm *et al.*, 2017; Goldfarb *et al.*, 2019; Noack *et al.*, 2019): hippocampus, parahippocampal gyrus, amygdala, cingulate cortex, thalamus, nucleus. caudatus, nucleus. accumbens and putamen. We applied Benjamini–Hochberg corrections (Benjamini and Hochberg, 1995; Nichols *et al.*, 2017) to account for increases in false discovery rate and report uncorrected *P*-values as well as corrected significance thresholds. ROI analyses were performed using fslmaths and featquery. We created masks in which each voxel was assigned a 1 that had a ≥ 50.0% chance of belonging to the specific ROI and then we binarized these masks. Subsequently, mean beta values (extracted from second-level analysis) were exported to SPSS and *post hoc* one-way ANOVAs were computed with sex as fixed factor and cortisol increase as covariate.

## Results

### Sex differences in cortisol, psychological and heart rate responses

**Fig. 2.**
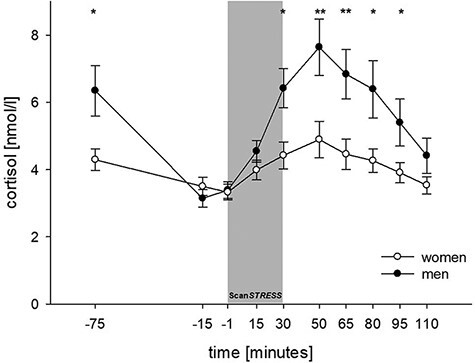
Salivary cortisol responses to Scan*STRESS* in women and men (±SEM). **P ≤ 0.01 and *P ≤ 0.05 indicate significant results of *post hoc* unpaired *t*-tests for each time point.

For cortisol measures, we detected a significant time × sex interaction (*F*_3,162_ = 3.33, *P *= 0.028, η^2^ = 0.045; see Figure 2) as well as significant main effects for time (*F*_3,162_ = 9.85, *P *≤ 0.001, η^2^ = 0.132) and sex (*F*_1,65_ = 6.69, *P* = .012, η^2^ = 0.093). While men showed significantly higher cortisol levels than women briefly after entering the lab (−75 min), levels subsequently decreased and both groups showed similar cortisol concentrations immediately prior to stress onset (−15 min). In response to stress exposure, men showed significantly higher cortisol responses than women. Results of calculated *post hoc t*-tests regarding each time point and cortisol increases are shown in Supplementary Results Table SB1. When analyzing the two subsamples separately, the main effect time reached significance in women (*F*_2,73_ = 3.47, *P *= 0.028, η^2^ = 0.104) and men (*F*_2,82_ = 8.01, *P* ≤ 0.001, η^2^ = 0.188). We detected 32.3% responders in women (all using OCs; 67.7% non-responders) and 77.8% responders in men (22.2% non-responders). Moreover, the timing of the individual cortisol peak was not significantly different between women and men (χ^2^(2) = 1.021, *P* = 0.600).

Consistent to our previous analysis (Henze *et al.*, 2020), we found significant main effects for time in affect measures and mean heart rate levels (*p*s ≤ 0.001, η^2^ > 0.299). Positive affect scores decreased and negative affect scores increased during Scan*STRESS*. Participants showed elevated heart rates during the stress blocks compared to the control blocks in both runs. We detected neither significant interactions of time × sex nor main effects sex regarding affect or heart rate measures (*p*s ≥ 0.153, η^2^ < 0.020). Given the absence of sex differences in affect and heart rate reactions, no sex-specific associations with neural responses were analyzed.

### Sex-specific associations of cortisol and neural responses

**Fig. 3.**
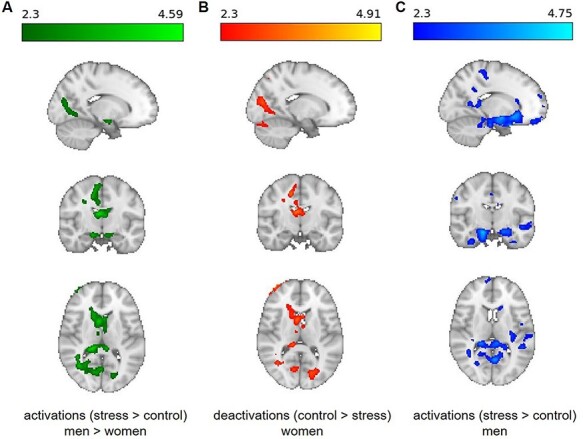
(A) Sex-specific cluster in an unpaired two-group difference analysis with continuous covariate interaction (grand mean centered, two-tailed combined FWE-corrected *P* < 0.05) describing a sex-specific relationship (men > women) between cortisol increases and neural responses (stress > control) in the hippocampus, parahippocampal gyrus, cingulate cortex, thalamus and nucleus. caudatus. (B) In women, higher cortisol increases were associated with more deactivation (control > stress) within cluster including the cingulate cortex, thalamus and nucleus. caudatus. (C) In men, higher cortisol increases were associated with more activation (stress > control) in a cluster comprising the hippocampus, parahippocampal gyrus, amygdala, cingulate cortex, prefrontal areas, nucleus. caudatus and nucleus. accumbens.

A whole-brain unpaired two-group difference analysis revealed neither significant sex-specific cluster for activations (stress > control) nor deactivations (control > stress); two-tailed combined FWE-corrected *P* < 0.05. However, when cortisol increases were used as covariate in a whole-brain unpaired two-group difference analysis with continuous covariate interaction (grand mean centered, two-tailed combined FWE-corrected *P* < 0.05), a sex-specific cluster reached significance (see Figure 3). In detail, we detected a sex-specific relationship between cortisol increases and neural responses within a cluster comprising the bilateral hippocampus, parahippocampal gyrus, (anterior and posterior) cingulate cortex, thalamus and nucleus. caudatus. When men were compared to women in the total sample, higher cortisol increases were found to be related to more activation within this cluster (see Figure 3A). In the female subsample alone, higher cortisol increases were associated with more deactivation in cingulate cortex, thalamus and nucleus. caudatus (see Figure 3B), while in the male subsample higher cortisol increases were associated with more activation in hippocampus, parahippocampal gyrus, amygdala, cingulate cortex, prefrontal areas, nucleus. caudatus and nucleus. accumbens (see Figure 3C). Peak voxels within the cluster are reported in the Supplementary Results Tables SB2-SB4.

**Fig. 4.**
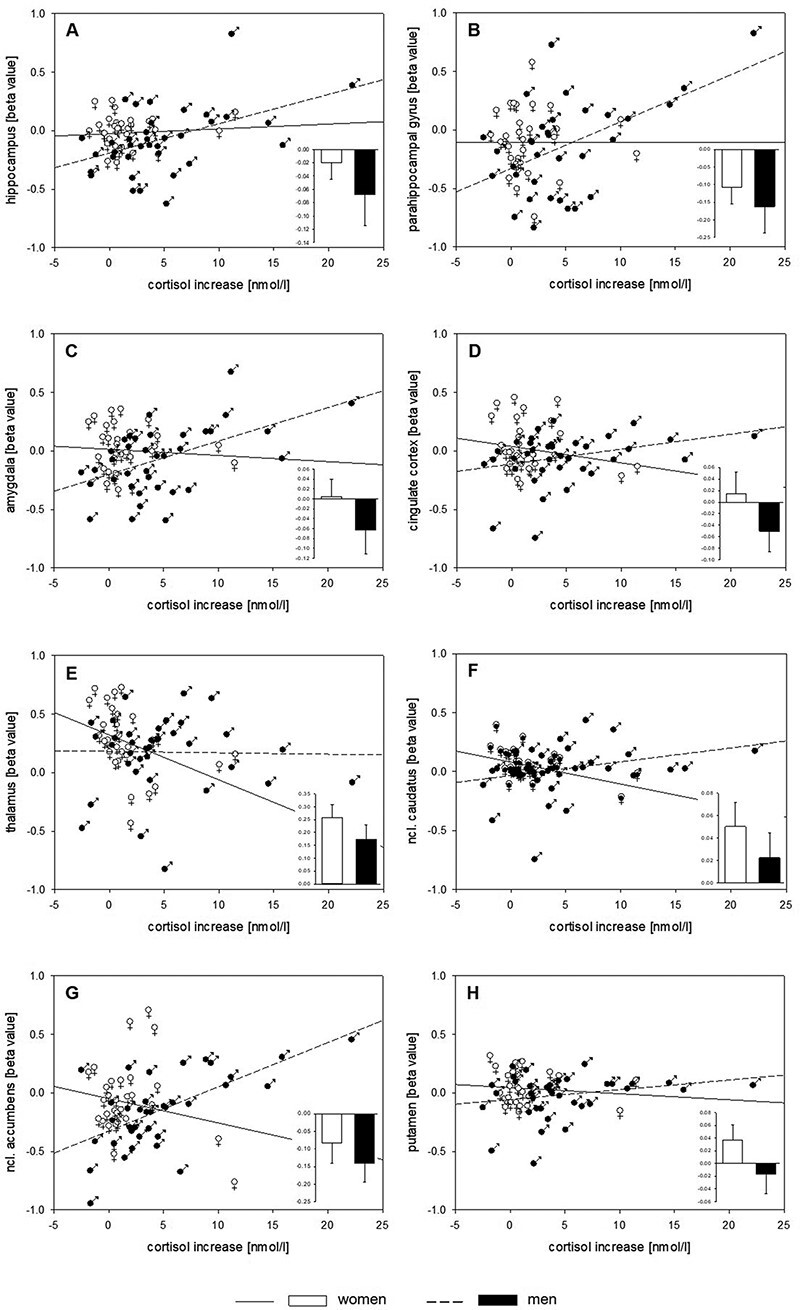
Sex-specific mean neural responses (±SEM, stress > control) and correlations of cortisol increase with beta values of the main task effect stress > control in the (A) hippocampus, (B) parahippocampal gyrus, (C) amygdala, (D) cingulate cortex, (E) thalamus, (F) nucleus. caudatus, (G) nucleus. accumbens and (H) putamen derived from masks using the Harvard Oxford Atlas.

Table 1 depicts the results from *post hoc* ROI analyses including uncorrected *P*-values and Benjamini–Hochberg-corrected significance thresholds. We found significant interactions of sex × cortisol increase for amygdala, nucleus. caudatus and nucleus. accumbens. Men showed positive associations between beta values and cortisol increases, while women showed negative associations (see Figure 4). Main effects of sex were found for hippocampus, parahippocampal gyrus, amygdala, cingulate cortex, nucleus. caudatus, nucleus. accumbens and putamen. *Post hoc* analyses in the two subsamples separately are displayed in Table 2. In women, we found negative associations between cortisol increases and beta values for thalamus and nucleus. caudatus. In men, positive associations emerged for hippocampus, parahippocampal gyrus, amygdala, cingulate cortex, nucleus. caudatus and nucleus. accumbens. It should be noted that these correlations remained significant after exploratory exclusion of a male participant showing a pronounced (but endocrinologically plausible) cortisol increase of 22.36 nmol/l (see Figure 4).

**Table 1.** Results from *post hoc* one-way ANOVAs with ‘sex’ as a fixed factor and ‘cortisol increase’ as a covariate for the hippocampus, parahippocampal gyrus, amygdala, cingulate cortex, thalamus, nucleus. caudatus, nucleus. accumbens and putamen, including uncorrected *P*-values and Benjamini–Hochberg-corrected significance thresholds (*n* = 8 ROIs)

ROI	Effect	Uncorrected values				
		degrees of freedom (df)	*F*	*P*-value	η^2^	Corrected significance threshold
Hippocampus	Sex × cortisol increase	1, 63	2.04	0.158	0.031	≤0.04375
	Sex	**1, 63**	**6.57**	0.013*	**0.094**	≤0.01875
	Cortisol increase	1, 63	3.93	0.052	0.059	≤0.00625
Parahippocampal gyrus	Sex × cortisol increase	1, 63	3.10	0.084	0.049	≤0.03125
	Sex	**1, 63**	**4.82**	0.032*	**0.074**	≤0.0375
	Cortisol increase	1, 63	3.01	0.088	0.048	≤0.01875
Amygdala	Sex × cortisol increase	1, 63	4.42	0.039*	0.066	≤0.01875
	Sex	**1, 63**	**9.46**	0.003*	**0.131**	≤0.0125
	Cortisol increase	1, 63	2.10	0.152	0.032	≤0.025
Cingulate cortex	Sex × cortisol increase	1, 63	3.35	0.072	0.051	≤0.025
	Sex	**1, 63**	**5.52**	0.022*	**0.081**	≤0.025
	Cortisol increase	1, 63	0.01	0.924	0.000	≤0.05
Thalamus	Sex × cortisol increase	1, 63	2.98	0.089	0.046	≤0.0375
	Sex	1, 63	2.19	0.144	0.034	≤0.05
	Cortisol increase	1, 63	3.38	0.071	0.052	≤0.0125
nucleus. caudatus	Sex × cortisol increase	1, 63	6.55	0.013*	0.094	≤0.0125
	Sex	**1, 63**	**4.96**	0.030*	**0.073**	≤0.03125
	Cortisol increase	1, 63	0.35	0.557	0.005	≤0.0375
nucleus. accumbens	Sex × cortisol increase	**1, 63**	**8.66**	0.005*	**0.121**	≤0.00625
	Sex	**1, 63**	**10.12**	0.002*	**0.138**	≤0.00625
	Cortisol increase	1, 63	0.075	0.391	0.012	≤0.03125
Putamen	Sex × cortisol increase	1, 63	1.41	0.240	0.022	≤0.05
	Sex	1, 63	4.17	0.045*	0.062	≤0.04375
	Cortisol increase	1, 63	0.07	0.798	0.001	≤0.04375

*Note*: * indicates significant results (*P* ≤ 0.05) and values in bold indicate significant results after Benjamini–Hochberg corrections.

**Table 2.** Results from *post hoc* correlation analyses between mean beta values of the hippocampus, parahippocampal gyrus, amygdala, cingulate cortex, thalamus, nucleus. caudatus, nucleus. accumbens and putamen with cortisol increase in the two subsamples separately including uncorrected *P*-values and Benjamini–Hochberg-corrected significance thresholds (*n* = 8 ROIs)

	Correlation with cortisol increase					
	Women (*n* = 31)			Men (*n* = 36)		
	Uncorrected values			Uncorrected values		
ROI	*r*	*P*-value	Corrected significance threshold	*r*	*P*-value	Corrected significance threshold
Hippocampus	0.087	0.321	≤0.0375	**0.466**	0.002*	≤0.025
Parahippocampal gyrus	−0.003	0.494	≤0.05	**0.510**	0.001*	≤0.0125
Amygdala	−0.079	0.336	≤0.04375	**0.516**	0.001*	≤0.0125
Cingulate cortex	−0.196	0.145	≤0.01875	0.311	0.032*	≤0.03125
Thalamus	−0.395	0.014*	≤0.0125	−0.020	0.456	≤0.05
nucleus. caudatus	**−0.449**	0.006*	≤0.00625	0.290	0.043*	≤0.0375
nucleus. accumbens	−0.193	0.149	≤0.025	**0.621**	0.001*	≤0.00625
Putamen	−0.116	0.266	≤0.03125	0.234	0.085	≤0.04375

*Note*: * indicates significant results (*P* ≤ 0.05) and values printed in boldface indicate significant results after Benjamini–Hochberg correction.

### Explorative analysis of sex differences in exposure-time effects

**Fig. 5.**
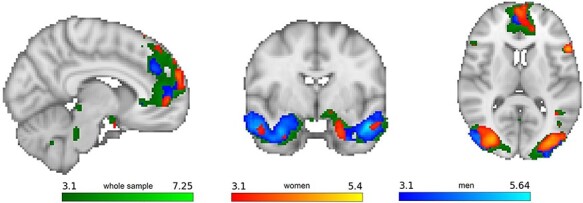
Activation changes over the two runs of Scan*STRESS* of the female (red to yellow) and male (blue to light blue) subsample compared to the total sample as reference [green to light green; (Henze *et al.*, 2020)].

As the research questions of the present study arose from findings of our aforementioned study (Henze *et al.*, 2020), we also addressed the question if women and men show distinct neural reactions in response to the two runs of Scan*STRESS*. A whole-brain two-way mixed effects ANOVA (two groups, two runs per subject, *z* > 3.1, FWE-corrected *P* < 0.05) did not reveal a significant run × group interaction. We found similar clusters in women and men comprising hippocampus, parahippocampal gyrus, amygdala, prefrontal cortex and cingulate cortex to respond differently to the two runs. Figure 5 illustrates these activation changes in women and men; peak voxels are reported in the Supplementary Results Tables SB5 and SB6. Consistent to our previous analysis (Henze *et al.*, 2020), post hoc ROI analyses (repeated measures ANOVAs, run as within-subjects factor and sex as between-subjects factor) revealed main effects of run for hippocampus, parahippocampal gyrus and amygdala (*p*s ≤ 0.001, η^2^ > 0.160). While we did not find significant interactions of run × sex (*p*s ≥ 0.367, η^2^ < 0.014), for thalamus, a significant main effect sex (*F*_1,61_ = 5.22, *P* = 0.026, η^2^ = 0.079) was detected, indicating mean response differences of women and men in the first run (women: *M* = −0.01, s.d. = 0.22; men: *M* = −0.10, s.d. = 0.26) compared to the second run (women: *M* = 0.01, s.d. = 0.21; men: *M* = −0.15, s.d. = 0.27).

## Discussion

### Sex-specific associations of cortisol and neural responses

The present data confirmed the well-known sex-specific cortisol stress response pattern, with men exhibiting higher responses than women (Nicolson *et al.*, 1997; Seeman *et al.*, 2001; Kudielka and Kirschbaum, 2005; Goel *et al.*, 2014; Liu *et al.*, 2017; Zänkert *et al.*, 2019). Higher cortisol levels in men occurred already 75 min prior to stress onset, suggesting a more pronounced anticipation response (Kirschbaum *et al.*, 1992; Kudielka and Kirschbaum, 2005). After the relaxation phase, women and men reached similar mean levels (see Figure 2). It should be noted again that all female participants used OCs and that women tested in the luteal phase of their menstrual cycles were repeatedly found to show higher cortisol responses to stress (Kirschbaum *et al.*, 1999; Rohleder *et al.*, 2001, 2003; Wolf *et al.*, 2001; Uhart *et al.*, 2006). In addition, it appears likely that the block design of Scan*STRESS*, with frequent interruptions of the stress induction by control blocks, might have hampered even higher cortisol responses. However, not using an alternating block design would cause other fMRI-related problems (e.g. limited detection power; Quaedflieg *et al.*, 2013; Noack *et al.*, 2019; Sandner *et al.*, 2020).

While empirical evidence for consistent interactions of distinct stress domains remains scarce (Cohen *et al.*, 2000; Campbell and Ehlert, 2012; Henze *et al.*, 2020), this is the first study focusing on sex-related differences between associations of cortisol and task-related neural responses to psychosocial stress. Whereas previous findings on sex-specific neural stress responses suggest pronounced striato-limbic activation in women and stronger frontal activation in men (Wang *et al.*, 2007; Seo *et al.*, 2011, 2017; Kogler *et al.*, 2015; Goldfarb *et al.*, 2019), our data revealed no such clear functional distinction for particular neural structures. Instead, we found different associations for women and men between cortisol reactions and responses of identical striato-limbic structures. A whole-brain analysis in the total sample documented higher cortisol increases in men to be associated with more activation in hippocampus, parahippocampal gyrus, cingulate cortex, thalamus and nucleus. caudatus compared to women. In the female subsample, higher cortisol increases were related to deactivations in these structures, whereas in the male subsample, higher cortisol increases were related to activations. In contrast to previous findings, proposing a small degree of overlap between the stress networks of women and men (Wang *et al.*, 2007; Seo *et al.*, 2011, 2017; Kogler *et al.*, 2017), our data corroborate differential responses for women and men in identical structures (Kogler *et al.*, 2016; Goldfarb *et al.*, 2019). ROI analyses confirmed interactions of sex × cortisol increase for amygdala, nucleus. caudatus and nucleus. accumbens, underpinning positive associations in men and negative associations in women, with the latter describing lower cortisol increases to be associated with more activation. Thus far, only resting state data exist, including two male-only samples (Veer *et al.*, 2011; Vogel *et al.*, 2015) and one mixed sample (Kogler *et al.*, 2016), reporting a positive effect pattern for men and a negative one for women.

The only structure that reached significance in whole brain as well as every ROI-based correlation analysis, also in both subsamples separately, is the nucleus. caudatus. As part of the striatum, this area showed sex-specific FC patterns partly depending on menstrual cycle phase (Yoest *et al.*, 2018; Hidalgo-Lopez *et al.*, 2020). Moreover, evidence for larger gray matter volumes in female brains within this structure exists (Luders *et al.*, 2009). Another stress-relevant area within the striatum is the nucleus. accumbens (McEwen *et al.*, 2016). As reward-related area, an expressed desire of revenge in men was found to be correlated with increased activity, while in women this was associated with deactivations (Singer *et al.*, 2006; Dumais *et al.*, 2018). In this context, two aspects should be considered: first, when applying Scan*STRESS*, participants are instructed to show maximal effort, consistent with the cover story that the study aims at investigating brain activations during maximal mental performance. Second, after the first run of Scan*STRESS*, participants are exposed to a standardized negative feedback regarding their performance combined with the urgent request to try harder. Therefore, these factors of psychosocial stress might have led to pronounced reactions of the nucleus. accumbens in particular and to an overall striatal response. Moreover, another Scan*STRESS* study that found associations between striatal activation and perceived group discrimination in ethnic minority individuals strengthen the evidence for an involvement of the striatum and inherent structures (Akdeniz *et al.*, 2014). While this view is also supported by a recent study that used a psychosocial stress paradigm (Kogler *et al.*, 2015), another study did not report sex differences in putamen responses during stress perception (Wang *et al.*, 2007). Therefore, it is tempting to speculate that the striatal network modulates sex-specific interactions in response to the repeated experience of failure and social-evaluative threat as induced by Scan*STRESS*. The finding of altered left amygdala FC to striatal regions correlating positively with cortisol in men but negatively in women (Kogler *et al.*, 2016) emphasizes this hypothesis. Moreover, a previously reported analysis of our present data on the association between cortisol and neural stress responses, independent of sex, revealed no significance for striatal structures (Henze *et al.*, 2020). This is consistent with the assumption of a sex-specific modulating striatal effect on stress responses.

Among others, the hippocampus has been considered as decisive HPA-axis-related structure ever since (Herman *et al.*, 2005; Jankord and Herman, 2008; Hermans *et al.*, 2014) and one of the most prominent findings describes deactivations in response to psychosocial stress along with negative associations with cortisol (Pruessner *et al.*, 2008). However, data exist showing the opposite (Noack *et al.*, 2019; Henze *et al.*, 2020). Here, we found a positive correlation of activations and cortisol in men confirming sex-related differences after stress induction for the hippocampus (Seo *et al.*, 2011; Yagi and Galea, 2019). Moreover, our data showed a comparable pattern regarding the parahippocampal gyrus for men, while in women no significant association with cortisol emerged for hippocampus and parahippocampal gyrus.

Concerning the amygdala and cingulate cortex, the dissociation between women and men is more obvious. Especially, for the amygdala, its activating impact on HPA axis responses to stress has been reported frequently (Herman *et al.*, 2005; Jankord and Herman, 2008; Noack *et al.*, 2019; Henze *et al.*, 2020). Previous work showed an association between stress and amygdala activation only in women (Wang *et al.*, 2007; Kogler *et al.*, 2015). We found that activations were associated with higher cortisol increases in men and lower values in women, confirming a sex-specific effect pattern regarding amygdala FC in association with cortisol (Kogler *et al.*, 2016). Moreover, this study also revealed a negative association of altered left amygdala FC with the anterior cingulate cortex in women, while the opposite was reported in a male-only sample (Veer *et al.*, 2012).

A negative association with cortisol was found for the thalamus in women, while in men no significant relationship emerged, confirming previous findings (Wang *et al.*, 2007). The thalamus is thought to actively and dynamically gate salient inputs, minimizing the importance of currently irrelevant ones (Wolff and Vann, 2019). A recent study showed altered thalamic network centrality in response to acute psychosocial stress within a male-only sample (Reinelt *et al.*, 2019). Moreover, previous studies have shown stress-driven changes in thalamic activation in women and men (Noack *et al.*, 2019). With reference to the aforementioned hypothesis on striatal involvement in sex-specific cortisol responses, the thalamus as adjacent structure may act as an additional coordinator. Nevertheless, there exists just as much evidence for pronounced thalamo-striatal stress reactions in women (Wang *et al.*, 2007) as in men (Seo *et al.*, 2011, 2017).

In sum, we found a dissociation between women and men regarding the association between neural and cortisol responses to acute psychosocial stress induction. Currently, it can only be speculated why these associations point in opposite directions. It appears conceivable that our results are consistent with the idea that sex-specific differences in the brain may indicate compensation mechanisms aimed at maintaining comparable abilities or preventing maladaptive differences (Grabowska, 2017). Hence, men might show a positive association between cortisol and striato-limbic responses and women a negative association to maintain similar and adaptive outcomes, e.g. comparable subjective and heart rate responses.

### Explorative analysis of sex differences in exposure-time effects

Although there is evidence that women and men respond differently to ongoing stress exposure (McEwen and Milner, 2017; Goldfarb *et al.*, 2019), we did not detect any significant sex-specific changes when comparing responses of the first with those of the second run of Scan*STRESS*. While increasing deactivations emerged for both subsamples, our results may, on a descriptive level, suggest different extents for women and men regarding the targeted clusters (see Figure 5).

### Heart rate and psychological responses

As previously reported (Henze *et al.*, 2020), we found a significant decline in positive affect ratings and an increase in reported negative affect. However, we did not detect significant sex differences. The fact that other studies reported different affect responses in women and men may be explainable by different affect measurements (e.g. visual analogue scales; Kelly *et al.*, 2006, 2008; Goldfarb *et al.*, 2019). Moreover, women and men exhibited similar heart rate responses during stress. In this regard, again, the composition of the present sample has to be considered. Earlier research supports the idea of a pronounced impact of menstrual cycle phases and/or OC use on the presence or absence of sex differences (Yoest *et al.*, 2018; Hidalgo-Lopez *et al.*, 2020; Sharma *et al.*, 2020), especially regarding psychological measures (Childs *et al.*, 2010; Albert *et al.*, 2015; Lewis *et al.*, 2019). Moreover, we generally assume that the basic characteristics of an fMRI block design—as mentioned earlier—interfere with even more pronounced responses. Hence, the overall lower stress intensity, achievable by scanner paradigms compared to laboratory stressors, has to be considered. Furthermore, the absence of sex differences in a particular outcome should not lead to the misconception that the neural substrates underlying these mechanisms are necessarily identical for women and men (Cahill, 2006; Goldfarb *et al.*, 2019).

## Limitations and conclusion

First, we have to acknowledge a certain limitation of the generalizability of our findings, as only university students participated in the present study. Nevertheless, our data suggest sex-specific cortisol reactions to be differentially associated with striato-limbic responses to psychosocial stress (Seo *et al.*, 2017). From a general perspective, we assume that detectable differences in stress responses between women and men are only partly due to biological sex (Juster *et al.*, 2016; Rich-Edwards *et al.*, 2018). It might be a fruitful approach to explicitly take sociocultural gender into account in future studies. Moreover, as our study sample included only OC-taking women, we have to emphasize that the present data may only contribute to a better understanding of differences in the association of neural stress responses and cortisol increases of women taking OCs *vs* men. It could well be appropriate to limit our conclusions to the (large) subgroup of women taking hormonal contraceptives, as it was previously found in female-only studies that OC use and menstrual cycle phase can influence the brain’s response to negative stimuli (Goldstein *et al.*, 2010; Petersen and Cahill, 2015) and psychosocial stress (Albert *et al.*, 2015; Chung *et al.*, 2016b). Furthermore, at least cortisol increases are known to be modulated not only by OCs but also by menstrual cycle phases (Kirschbaum *et al.*, 1999; Zänkert *et al.*, 2019). However, as simple group-level analyses contrasting female and male neural responses to psychosocial stress paradigms failed to reveal consistent differences (in the present as well as in previous studies: Kogler *et al.*, 2015; Chung *et al.*, 2016a; Dahm *et al.*, 2017; Kogler *et al.*, 2017), it appears unlikely that corresponding differences between OC-taking and naturally cycling women are extremely large. To date, studies on the impact of sex on the interaction between cortisol and neural stress responses in OC-taking women, in women in luteal and follicular phase as well as in men do not exist.

Even though women and men differ in their overall stress reactivity and regarding the prevalence rates of certain stress-related pathologies, our findings do not support the view of a clear neuroanatomically differentiable ‘female-typical’ and ‘male-typical’ response to stress. Instead, our data provide further evidence for the idea that considering complex interactions and quantitative variables such as individual cortisol increases is a more suitable approach to elucidate sex-related differences in central stress regulation (Shalev *et al.*, 2020).

## Supplementary Material

nsab062_Supp
