# Supplementary material for: Sex-specific interaction between cortisol and striato-limbic responses to psychosocial stress
Source: Soc Cogn Affect Neurosci. 2021 Apr 16;16(9):972–84. doi: 10.1093/scan/nsab062 (PMC8421693; doi:10.1093/scan/nsab062)
Supplement: nsab062_Supp [file nsab062_supp.zip › Supplemental_Information_Henze.docx]

**Sex-specific interaction between cortisol and striato-limbic responses to psychosocial stress**

***Supplemental Information***

**A Supplemental Methods**

**A.1 – Description of Scan*STRESS***

The stress induction includes several psychosocial components such as pressure to perform, time pressure, forced failure, social-evaluative threat, uncontrollability, and unpredictability. Participants receive the instruction that the aim of the study is the “investigation of brain activation during maximal mental performance”. Thus, they are asked “to show maximal effort”.

Scan*STRESS* was implemented in Presentation^®^ software (Version 12.9, www.neurobs.com). It consists of two different tasks presented in a block design via monitor. The first task prompts the participant to match a three-dimensional figure to its rotated equivalent from three options presented below the target figure; source of stimulus material: (Peters & Battista, 2008). Analog to the Trier Social Stress Test (TSST; (Kirschbaum et al., 1993)) the second task asks the participant to continuously subtract a number (here 13) from a four-digit number. The correct answer is presented below together with three other response options. In case of an error, participants have to start subtracting all over again. In the stress blocks, the participant has to perform under time pressure presented by a countdown timer, signaling the remaining time. Both, task speed as well as difficulty are adapted by the participant’s individual performance to ensure frequent failure. In control conditions, tasks are less demanding and performed without time pressure and social evaluation (number matching without subtraction and figure matching without rotation). Answers are given with a five-button hand-shaped response box.

The block design of Scan*STRESS* (two runs of 680 seconds duration each) includes repeated 60 seconds task blocks (control or stress) preceded by five seconds announcement and followed by 20 seconds rest period. In sum, the paradigm comprises 16 epochs of 60 seconds each with alternating stress and control blocks presented in two runs (see Figure A.1.B).

In the present study, Scan*STRESS* was applied as previously reported (Streit et al., 2014) but with a slightly modified protocol. Participants arrived at the laboratory 75 minutes prior to stress onset (see Figure A.1.A). After collection of the first saliva sample, a prolonged (45 minutes) relaxing phase was implemented. During this phase, participants watched a neutral movie (nature documentary) while resting in an armchair. Forty-five minutes prior to stress, participants received a sugary drink (75 g glucose in 200-mL herbal tea) to facilitate cortisol reactivity (Gonzalez-Bono et al., 2002; Kirschbaum et al., 1997; Zänkert et al., 2020). During relaxation, the participants completed a brief training session of the Scan*STRESS* control blocks (approximately 25 minutes prior to stress onset)*.* To achieve a more abrupt passage from relaxation to stress exposure, the duration of the participant’s transition into the scanner tube was optimized (< 10 minutes) and the observation panel was presented to the participant immediately before paradigm onset. After immersion into the scanner tube (time point -15 minutes) and subsequent pre-measurements (localizer etc.), participants were confronted with the demanding tasks immediately after receiving the instructions of the panel via live video stream and audio system (time point -1 minute). The observation panel consisted of a female and a male researcher in professional attire, sitting in the control room. Additionally, the panel informed the participant that behavior, mimics, and answers will be monitored via live video transmission of the participants face. Also, a live video transmission of the panel was presented during the scanning procedure to induce social-evaluative threat. During the task blocks, the panel explicitly gazed at the monitors. The observers gave standardized disapproving visual feedback after wrong or slow answers by pressing buttons on a “buzzer” visible on the video transmission. Depending on the individual performance and the button pressed, the participants either received the message “Error!” or the demand “Work faster!”. In the control blocks, the panel behaved passively, gave no feedback, and turned away from the camera (but was still visible for the participant). A diagonal grey cross signaling that no observation takes place overlayed the video picture (see Figure A.1.C). After the first run (time point +15 minutes), a saliva sample was collected and the experimenter announced that the panel was unsatisfied with the participant’s performance. Thereafter, one of the panel members notified the participant that the performance was below average and that the participant has to improve in the second run, otherwise the fMRI data will be useless.

After completion of the fMRI scans (65 minutes in total, time point +65 minutes) that also included a multiband resting state sequence (results not reported in the present manuscript) as well as anatomical measurements, participants remained in the laboratory to fill out questionnaires (time point +65 – +110 minutes). Finally, they received a detailed debriefing.

Food consumption or drinking (except water) was not allowed during the whole procedure. Regarding the time prior to arriving at the laboratory, participants were asked not to consume major meals 90 minutes before protocol onset (i.e., three hours prior to stress onset). Participants arrived at standardized times (12:00 or 01:20 PM) and scan onset was not before 01:20 PM.


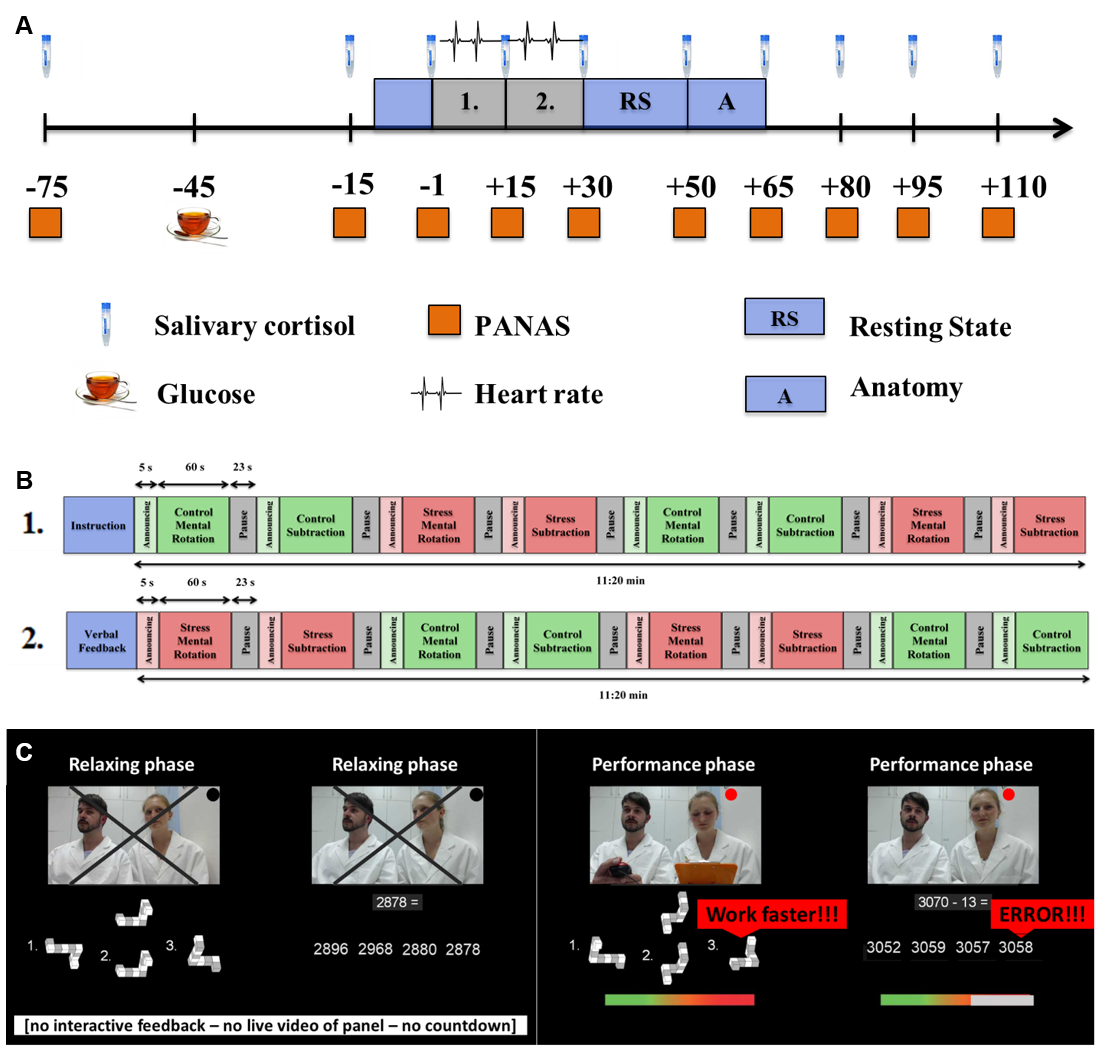


**Figure A.1.** A) Visualization of the experimental procedure including cortisol, psychological, and heart rate measures. B) Design of Scan*STRESS* with two runs, preceded by an instruction phase and interrupted by negative verbal feedback of the panel. C) Screenshot of the two different conditions (control and stress) and the two different tasks for each condition.

**A.2 – Salivary cortisol analysis**

In order to determine the cortisol concentration in the saliva sample we used a time-resolved fluorescence immunoassay. The saliva samples were stored at -20 °C until analysis. After thawing, saliva samples were centrifuged at 2000 g for six minutes, which resulted in a clear supernatant of low viscosity. 100 µl of saliva were used for duplicate analysis (50 µl per well). Cortisol levels were determined employing a competitive solid phase time-resolved fluorescence immunoassay with fluorometric end-point detection (DELFIA). 96-well-Maxisorb microtiterplates (Nunc) were coated with swine-anti-rabbit immunoglobulin. After an inkubation period of 48 hours at 4° C plates were washed three times with washbuffer (pH = 7,4; contains sodiumphosphat and the Tween-40). In the next step, the plates were coated with a rabbit anti-cortisol antibody and incubated for 48 hours at 4° C. Synthetic saliva mixed with cortisol in a range from 0-100 nmol/L served as standards. Standards, controls (saliva pools) and samples were given in duplicate wells. 50 µl of biotin-conjugated cortisol was added and after 30 minutes of incubation the non-binding cortisol/biotin-conjugated cortisol was removed by washing (three times). 200 µl europium-streptavidin (Perkin Elmer, Rodgau, Germany) was added to each well and after 30 minutes and six times of washing 200 µl enhancement solution was added (Pharmacia, Freiburg, Germany). Within 15 minutes on a shaker, the enhancement solution induced the fluorescence which can be detected with a VICTOR™ X4 Multilabel Plate Reader (Perkin Elmer, Massachusetts, USA). With a computer-controlled program, a standard curve was generated and the cortisol concentration of the samples were calculated. The intra-assay coefficient of variation was between 4.0% and 6.7%, and the corresponding inter-assay coefficients of variation were between 7.1% and 9.0%. The detection limit of the assays is 0.179 nmol/L.

**Table A.3**

*Skewness and kurtosis parameters of each measurement time point of cortisol.*

|  |  | skewness (± SEM) | kurtosis (± SEM) |
| --- | --- | --- | --- |
| -75 minutes | women | 1.854 (± .421) | 5.299 (± .821) |
|  | men | 1.866 (± .393) | 3.244 (± .768) |
| -15 minutes | women | 1.301 (± .421) | 1.261 (± .821) |
|  | men | 1.441 (± .393) | 1.911 (± .768) |
| -1 minute | women | .684 (± .421) | - .403 (± .821) |
|  | men | .918 (± .393) | .708 (± .768) |
| 15 minutes | women | 1.008 (± .421) | 1.549 (± .821) |
|  | men | .964 (± .393) | .359 (± .768) |
| 30 minutes | women | 1.069 (± .421) | 1.262 (± .821) |
|  | men | 2.126 (± .393) | 5.068 (± .768) |
| 50 minutes | women | 1.373 (± .421) | 1.695 (± .821) |
|  | men | 2.383 (± .393) | 7.149 (± .768) |
| 65 minutes | women | .727 (± .421) | - .308 (± .821) |
|  | men | 1.854 (± .393) | 2.863 (± .768) |
| 80 minutes | women | .513 (± .421) | .318 (± .821) |
|  | men | 1.804 (± .393) | 2.348 (± .768) |
| 95 minutes | women | .615 (± .421) | .138 (± .821) |
|  | men | 2.115 (± .393) | 4.352 (± .768) |
| 110 minutes | women | .222 (± .421) | - .623 (± .821) |
|  | men | 1.770 (± .393) | 2.693 (± .768) |
| increase | women | 2.221 (± .421) | 5.525 (± .821) |
|  | men | 1.455 (± .393) | 2.718 (± .768) |

**A.4 – FSL analysis**

Data were analyzed using Version 6.00 of FSL (FMRIB's Software Library, www.fmrib.ox.ac.uk/fsl, Oxford, UK). The first five echo-planar imaging images volumes were discarded to allow for T1 equilibration. FMRI data processing was carried out using FEAT version 6.0 (FMRI Expert Analysis Tool). Registration to high resolution structural and standard space images was carried out using FLIRT. Registration from high resolution structural to standard space was then further refined using FNIRT nonlinear registration. The following pre-statistics processing was applied: motion correction using MCFLIRT, slice-timing correction using Fourier-space time-series phase-shifting, non-brain removal using BET, spatial smoothing using a Gaussian kernel of FWHM 8.0 mm, grand-mean intensity normalization of the entire 4D dataset to Montreal Neurological Institute (MNI) 152 space by a single multiplicative factor, highpass temporal filtering (Gaussian-weighted least-squares straight line fitting, with sigma = 120.0 s). Time-series statistical analysis was carried out using FILM with local autocorrelation correction.

**B Supplemental Results**

**Table B.1**

*Comparison (mean ± SD) between women and men regarding cortisol levels [nmol/L] at each time point and cortisol increases [nmol/L].*

|  | women (*n* = 31) | men  (*n* = 36) | df | *t* | *p*-value | *d* |
| --- | --- | --- | --- | --- | --- | --- |
| -75 minutes | 4.41 (± 1.94) | 6.40 (± 4.61) | 48.49 | -2.36 | **.022** | .563 |
| -15 minutes | 3.64 (± 1.64) | 3.18 (± 1.58) | 62.68 | 1.16 | .251 | -.286 |
| -1 minute | 3.50 (± 1.38) | 3.43 (± 1.51) | 64.77 | .21 | .836 | -.048 |
| 15 minutes | 4.13 (± 1.77) | 4.51 (± 1.97) | 64.88 | - .82 | .413 | .203 |
| 30 minutes | 4.56 (± 2.49) | 6.23 (± 3.41) | 63.39 | -2.31 | **.024** | .559 |
| 50 minutes | 4.75 (± 2.99) | 7.19 (± 4.40) | 61.83 | -2.69 | **.009** | .649 |
| 65 minutes | 4.33 (± 2.18) | 6.67 (± 4.42) | 52.68 | -2.79 | **.007** | .671 |
| 80 minutes | 4.20 (± 1.75) | 6.33 (± 5.26) | 43.74 | -2.28 | **.028** | .543 |
| 95 minutes | 3.83 (± 1.46) | 5.35 (± 4.31) | 44.03 | -1.99 | .053 | .472 |
| 110 minutes | 3.50 (± 1.19) | 4.39 (± 3.21) | 45.74 | -1.53 | .132 | .368 |
| increase | 1.61 (± 2.29) | 4.90 (± 5.21) | 56.13 | -3.25 | **.002** | .818 |

*Note.* Significant *t*-tests are in boldface.

**Table B.2**

*Structures of a sex-specific (men > women) cluster in response to stress > control with cortisol increase as covariate (two-tailed combined test, FWE p < .05) including z- and p-values as well as the localization of peak voxels.*

| **structure** | | **statistics** | | | **MNI coordinates** | | |
| --- | --- | --- | --- | --- | --- | --- | --- |
|  |  | **K** | ***p*** | **z** | **X** | **Y** | **Z** |
| **cingulate gyrus, posterior division** | **right** | **9413** | **< .001** | **4.34** | **32** | **-56** | **0** |
|  |  |  |  | 4.28 | 26 | -46 | - 2 |
|  |  |  |  | 4.27 | - 6 | - 4 | 22 |
|  |  |  |  | 3.87 | 14 | -52 | 44 |
| thalamus |  |  |  | 4.01 | 16 | -36 | 8 |
|  |  |  |  | 4.00 | 4 | -14 | 18 |

*Note.* Global cluster maxima are in boldface.

**Table B.3**

*Significant cluster in the female subsample in response to control > stress associated with cortisol as covariate (two-tailed combined test, FWE p < .05) including z- and p-values as well as the localization of peak voxels.*

| **structure** | | **statistics** | | | **MNI coordinates** | | |
| --- | --- | --- | --- | --- | --- | --- | --- |
|  |  | **K** | ***p*** | **z** | **X** | **Y** | **Z** |
| **lingual gyrus** | **right** | **4081** | **< .001** | **3.92** | **28** | **-48** | **- 4** |
|  |  |  |  | 3.88 | 32 | -56 | - 2 |
|  |  |  |  | 3.47 | 14 | -70 | 2 |
| precuneus cortex |  |  |  | 3.85 | 14 | -52 | 46 |
|  |  |  |  | 3.24 | 10 | -64 | 48 |
| **cingulate gyrus, posterior division** | right | 2404 | < .001 | 3.64 | 14 | - 8 | 48 |
| thalamus |  |  |  | 3.61 | 6 | -14 | 18 |
| ncl. caudatus |  |  |  | 3.30 | 6 | 8 | 10 |
| cingulate gyrus, anterior division |  |  |  | 3.28 | 6 | - 2 | 28 |

*Note.* Global cluster maxima are in boldface.

**Table B.4**

*Significant cluster in the male subsample in response to stress > control associated with cortisol as covariate (two-tailed combined test, FWE p < .05) including z- and p-values as well as the localization of peak voxels.*

| **structure** | | **statistics** | | | **MNI coordinates** | | |
| --- | --- | --- | --- | --- | --- | --- | --- |
|  |  | **K** | ***p*** | **z** | **X** | **Y** | **Z** |
| **frontal orbital cortex** | **left** | **14564** | **< .001** | **4.75** | **-28** | **12** | **-24** |
|  |  |  |  | 4.35 | -18 | 12 | -28 |
|  |  |  |  | 4.14 | -20 | 18 | -14 |
| amygdala | right |  |  | 4.43 | 12 | - 6 | -18 |
| hippocampus | left |  |  | 4.08 | -22 | -18 | -16 |

*Note.* Global cluster maxima are in boldface.

**Table B.5**

*Activated structures in women during psychosocial stress (stress > control, control > stress) with run as regressor (two-tailed combined test, FWE p < .05) including z- and p-values as well as the localization of peak voxels.*

| **structure** | | **statistics** | | | **MNI coordinates** | | |
| --- | --- | --- | --- | --- | --- | --- | --- |
|  |  | **K** | ***p*** | **z** | **X** | **Y** | **Z** |
| **lateral occipital cortex, superior division** | **right** | **4402** | **< .001** | **5.4** | **28** | **-84** | **28** |
|  |  |  |  | 5.35 | 34 | -86 | 26 |
|  |  |  |  | 4.75 | 34 | -82 | 14 |
| occipital fusiform gyrus |  |  |  | 4.95 | 26 | -72 | -10 |
|  |  |  |  | 4.82 | 26 | -64 | -12 |
| temporal occipital fusiform cortex |  |  |  | 4.73 | 32 | -56 | -12 |
| **frontal pole** | **left** | **1714** | **< .001** | **4.55** | **- 8** | **48** | **44** |
|  |  |  |  | 4.28 | -10 | 54 | 40 |
|  |  |  |  | 4.27 | - 4 | 56 | 38 |
|  | right |  |  | 4.34 | 12 | 60 | 26 |
|  |  |  |  | 4.26 | 6 | 64 | 18 |
|  |  |  |  | 4.05 | 0 | 60 | 10 |
| **lateral occipital cortex, inferior division** | **left** | **1439** | **< .001** | **4.67** | **-38** | **-80** | **6** |
|  |  |  |  | 3.85 | -50 | -78 | -12 |
|  |  |  |  | 3.83 | -44 | -78 | -16 |
| lateral occipital cortex, superior division |  |  |  | 4.64 | -32 | -86 | 12 |
| occipital pole |  |  |  | 3.85 | -24 | -90 | 24 |
| **temporal pole** | **right** | **782** | **< .001** | **4.86** | **48** | **18** | **-26** |
|  |  |  |  | 4.66 | 54 | 10 | -32 |
| frontal orbital cortex |  |  |  | 3.95 | 32 | 18 | -20 |
|  |  |  |  | 3.87 | 28 | 20 | -24 |
| inferior temporal gyrus, anterior division |  |  |  | 3.38 | 50 | 0 | -38 |
| **middle temporal gyrus, posterior division** | **left** | **719** | **< .001** | **5.17** | **-52** | **-42** | **- 2** |
|  |  |  |  | 4.27 | -66 | -28 | -10 |
|  |  |  |  | 3.69 | -56 | -18 | -18 |
|  |  |  |  | 3.67 | -68 | -26 | -18 |
| superior temporal gyrus, posterior division |  |  |  | 3.74 | -66 | -18 | - 6 |
| **temporal occipital fusiform cortex** | **left** | **376** | **< .05** | **3.85** | **-28** | **-60** | **-14** |
|  |  |  |  | 3.57 | -26 | -54 | -18 |
| parahippocampal gyrus, superior division |  |  |  | 3.77 | -24 | -34 | -20 |
| occipital fusiform gyrus |  |  |  | 3.62 | -22 | -72 | -12 |
|  |  |  |  | 3.51 | -22 | -76 | -14 |
| **angular gyrus** | **left** | **331** | **< .05** | **4.43** | **-54** | **-56** | **26** |
| lateral occipital cortex, superior division |  |  |  | 3.25 | -44 | -64 | 16 |

*Note.* Global cluster maxima are in boldface.

**Table B.6**

*Activated structures in men during psychosocial stress (stress > control, control > stress) with run as regressor (two-tailed combined test, FWE p < .05) including z- and p-values as well as the localization of peak voxels.*

| **structure** | | **statistics** | | | **MNI coordinates** | | |
| --- | --- | --- | --- | --- | --- | --- | --- |
|  |  | **K** | ***p*** | **z** | **X** | **Y** | **Z** |
| **temporal pole** | **right** | **3226** | **< .001** | **5.28** | **48** | **18** | **-26** |
|  |  |  |  | 5.35 | 54 | 8 | -24 |
|  |  |  |  | 5.06 | 54 | 14 | -28 |
|  |  |  |  | 5.00 | 56 | 10 | -32 |
| middle temporal gyrus, anterior division |  |  |  | 5.37 | 52 | 4 | -22 |
| **middle temporal gyrus, posterior division** | **left** | **2668** | **< .001** | **5.64** | **-56** | **-16** | **-14** |
| temporal pole |  |  |  | 5.14 | -52 | 6 | -26 |
|  |  |  |  | 4.96 | -44 | 14 | -32 |
| **lateral occipital cortex, inferior division** | **right** | **2176** | **< .001** | **4.69** | **48** | **-80** | **4** |
|  |  |  |  | 4.66 | 46 | -68 | -10 |
|  |  |  |  | 4.37 | 34 | -86 | 10 |
| occipital pole |  |  |  | 4.42 | 24 | -94 | 26 |
|  |  |  |  | 4.38 | 28 | -92 | 28 |
| **paracingulate gyrus** | **left** | **1619** | **< .001** | **4.47** | **- 4** | **42** | **34** |
|  |  |  |  | 4.41 | 0 | 56 | 8 |
|  |  |  |  | 4.35 | 0 | 54 | 14 |
| superior frontal gyrus |  |  |  | 4.45 | - 2 | 44 | 50 |
|  |  |  |  | 4.42 | - 2 | 54 | 22 |
| frontal pole |  |  |  | 4.44 | - 8 | 46 | 44 |
| **lateral occipital cortex, superior division** | **left** | **1258** | **< .001** | **4.41** | **-36** | **-88** | **8** |
| lateral occipital cortex, inferior division |  |  |  | 4.29 | -46 | -80 | - 2 |
|  |  |  |  | 4.26 | -48 | -78 | - 8 |
|  |  |  |  | 3.94 | -34 | -82 | 0 |
| occipital pole |  |  |  | 4.08 | -26 | -94 | 8 |
|  |  |  |  | 3.90 | -30 | -98 | 10 |

*Note.* Global cluster maxima are in boldface.

**Supplemental References**

Gonzalez-Bono, E., Rohleder, N., Hellhammer, D. H., Salvador, A., & Kirschbaum, C. (2002) Glucose but not protein or fat load amplifies the cortisol response to psychosocial stress. Hormones and Behavior, 41, 328-333.

Kirschbaum, C., Pirke, K. M., & Hellhammer, D. H. (1993) The 'Trier Social Stress Test'--a tool for investigating psychobiological stress responses in a laboratory setting. Neuropsychobiology, 28, 76-81.

Kirschbaum, C., Gonzalez Bono, E., Rohleder, N., Gessner, C., Pirke, K. M., Salvador, A., & Hellhammer, D. H. (1997) Effects of fasting and glucose load on free cortisol responses to stress and nicotine. Journal of Clinical Endocrinology and Metabolism, 82, 1101-1105.

Peters, M., & Battista, C. (2008) Applications of mental rotation figures of the Shepard and Metzler type and description of a mental rotation stimulus library. Brain and Cognition, 66, 260-264.

Streit, F., Haddad, L., Paul, T., Frank, J., Schafer, A., Nikitopoulos, J., Akdeniz, C., Lederbogen, F., Treutlein, J., Witt, S., Meyer-Lindenberg, A., Rietschel, M., Kirsch, P., & Wüst, S. (2014) A functional variant in the neuropeptide S receptor 1 gene moderates the influence of urban upbringing on stress processing in the amygdala. Stress, 17, 352-361.

Zänkert, S., Kudielka, B. M., & Wüst, S. (2020) Effect of sugar administration on cortisol responses to acute psychosocial stress. Psychoneuroendocrinology, 115, 104607.
